# Galectin-3 and sST2 as Prognosticators for Heart Failure Requiring Extracorporeal Life Support: Jack n’ Jill

**DOI:** 10.3390/biom11020166

**Published:** 2021-01-27

**Authors:** Jianli Bi, Vidu Garg, Andrew R. Yates

**Affiliations:** 1 Center for Cardiovascular Research, Nationwide Children’s Hospital, Columbus, OH 43205, USA; jianli.bi@nationwidechildrens.org (J.B.); vidu.garg@nationwidechildrens.org (V.G.); 2 The Heart Center, Nationwide Children’s Hospital, Columbus, OH 43205, USA; 3 Department of Pediatrics, The Ohio State University, Columbus, OH 43205, USA; 4 Department of Molecular Genetics, The Ohio State University, Columbus, OH 43205, USA

**Keywords:** extracorporeal life support, mechanical circulatory support, ECMO, VAD, galectin-3, sST2, heart failure

## Abstract

Extracorporeal life support provides perfusion for patients with heart failure to allow time for recovery, function as a bridge for patients to heart transplantation, or serve as destination therapy for long term mechanical device support. Several biomarkers have been employed in attempt to predict these outcomes, but it remains to be determined which are suitable to guide clinical practice relevant to extracorporeal life support. Galectin-3 and soluble suppression of tumorigenicity-2 (sST2) are two of the more promising candidates with the greatest supporting evidence. In this review, we address the similarities and differences between galectin-3 and sST2 for prognostic prediction in adults and children with heart failure requiring extracorporeal life support and highlight the significant lack of progress in pediatric biomarker discovery and utilization.

## 1. Introduction

Heart failure is a life-threatening condition in both adults and children and is associated with high mortality, morbidity and cost of care. The incidence of heart failure in the general population is 2000/100,000 in adults [1,2] and 0.87–7.4/100,000 in children [3]. Extracorporeal life support (ECLS) including ventricular assist device (VAD) implantation and extracorporeal membrane oxygenation (ECMO) is required for patients with advanced or end-staged heart failure either as destination therapy or as a bridge-to-transplantation therapy. Over 25,000 adult and 21,000 children (including neonatal and pediatric patients) cases of ECLS were required globally, for cardiac indications, in the past 30 years. The overall survival rate was 59% in adults and 68% in children [4]. Limited literature is available to document prognostic markers for myocardial recovery in patients with refractory heart failure requiring circulatory support after decades of research. Early attempts to identify a biomarker to predict outcomes of ECLS have followed the evolution of cardiac biomarker testing utilizing brain natriuretic peptide (BNP) [5] and its N-terminal fragment, NT-proBNP [6] in the early 2000s through the recognition of cardiac troponin 10 years later [7]. The early decrease in BNP level is indicative of ventricular unloading during ECLS but the rebound in BNP level after decannulation suggests BNP is not an ideal biomarker to predict complete normalization of cardiac function [5]. Other heart failure related biomarkers which have been explored in patients who underwent ECLS include dynamic BNP [8], galectin-3 [9], ST2 [10], matrix metalloproteinase-9 (MMP-9) [11], tissue inhibitors of metalloproteinase-1 (TIMP-1) [11], MMP-2 [11,12], osteopontin [13], MR-proANP [14], proADM [14], and copeptin [14]. The above-depicted biomarkers may assist to predict outcomes of heart failure requiring ECLS under limited circumstances and their identifications are summarized in Table 1. Nevertheless, no single blood biomarker has demonstrated superiority to predict outcomes of heart failure requiring ECLS, but galectin-3 and ST2 have been promising and may be worthwhile to study further [15]. Unfortunately, a decade has passed with no significant progress in our ability to predict outcomes in patients with heart failure requiring ECLS. The failure of a single biomarker and/or single time-point measurement suggest that one may need to employ a combination of biomarkers with associated dynamic changes to predict outcomes in this context. Recently, there is growing interest in the use of galectin-3 and soluble suppression of tumorigenicity-2 (sST2) as potentially reliable prognostic markers [16]. These recent studies have demonstrated that sST2 provides independent predictive value beyond NT-proBNP and cardiac troponin for all-cause cardiovascular mortality in adult patients with chronic heart failure, which may be one explanation for this evolution [17]. Additionally, high levels of galectin-3 and BNP are often found before implantation of a ventricular assist device in patients with terminal heart failure, but elevated BNP failed to identify patients who would not survive VAD support. This prompted interest in galectin-3 levels which could better predict outcomes [18]. If proved in additional studies, the early prognostic value of gelactin-3 and sST2 to accurately identify patients destined for unfavorable recovery after ECLS implementation could provide a critical opportunity to modify treatment algorithms to a more personalized therapeutic approach to improve outcomes. Galectin-3 and sST2 are linked to the development of fibrosis which prevents recovery of myocardial function and may indicate severity of the disease state. In this review, we provide an overview of the recent clinical interpretation of galectin-3 and sST2 and emphasize their similarities and differences for the prognostic prediction of heart failure requiring ECLS. We also address the significant lack of data on galectin-3 and sST2 in pediatric patients undergoing ECLS and attempt to raise awareness about the novel utilization of galection-3 and sST2 as prognosticators in the pediatric population.

## 2. Galectin-3

Galectin-3 is a member of the galectins family of carbohydrate-binding proteins with specificity for *N*-acetyllactosamine (LacNAc)-containing glycoproteins, and the only known one with a single carbohydrate recognition domain and a unique *N*-terminus [19,20]. It is a 30 kDa molecule encoded by the LGALS3 gene that is located on chromosome 14, locus q21–q22 [21]. It is mainly secreted by macrophages and regulates basic cellular functions including growth, proliferation, differentiation and inflammation [22,23,24,25] and importantly has been found to play a role in cardiac fibrosis [26,27]. Evidence that links Galectin-3 to pathogenesis of heart failure has not been fully elucidated. However, recent studies have suggested that galectin-3 can help to predict prognosis of heart failure and adverse events in various clinical settings such as ST elevation myocardial infraction [28], congenital heart disease patients with a Fontan circulation [29] and survivors of out-of-hospital cardiac arrest [30]. In addition, its levels have correlated with morbidity and mortality in patients with heart failure [31,32,33,34]. Higher values (>15.3 ng/mL) of galectin-3 have been reported to show a correlation with the severity of heart failure [35].

VAD implantation is a standard ECLS modality for adult patients with end-staged heart failure. A retrospective study [36] including 57 adult patients with severe heart failure (NYHA Class IIIB–IV) who underwent VAD implantation found that a lower galectin-3 concentration (<30 ng) at the time of VAD implantation was associated with better prognosis when compared to an elevated concentration (>30 ng/mL) 2 years after VAD implantation. Similarly, the plasma galectin-3 concentration immediately before VAD implantation in patients who did not survive ECLS was significantly higher than that in those who were weaned from VAD support or received heart transplantation (18.8 ng/mL vs. 15.3 ng/mL). An additional study in adults noted that a higher galectin-3 concentration (>17 ng/mL) was associated with poor survival in low- or medium-risk VAD patients. However, the galectin-3 concentration was not a predictor in high-risk VAD patients [36]. These controversial results suggest that a single biomarker is limited in its ability to predict a clinically significant outcome, which is likely the result of multiple factors. A combination of the biomarkers may be required to eliminate this limitation. It is important to note that there is discrepancy in defining the clinically important cut-off values for galectin-3 in the above-mentioned studies, where the at-risk population was reported to be greater than 17 or 30 ng/mL [36,37]. The underlying reasons for this are unknown but may be related to differences in patient populations or techniques. Importantly, the galectin-3 concentrations were determined by different commercial kits in the studies above.

There is significantly less literature regarding galectin-3 in pediatric patients as compared to adults. Similar to adults, the galectin-3 concentration has been reported to be higher in children (median age: 9 years) with chronic heart failure than those (median age: 8.5 years) with normal heart function (9.46 ± 5.43 vs. 1.5 ± 0.66 ng/mL, *p* < 0.001). The increased galectin-3 concentration is associated with the severity of heart failure and can be reduced by spironolactone treatment [38]. The reduction of galectin-3 after spironolactone administration may be related to improvement of heart function. This suggests that galactin-3 may be used as a marker of disease severity in children with chronic heart failure and could potentially guide response to treatment in pediatric patients. In terms of the clinical value of galectin-3 for prognosis prediction in pediatric patients, a prospective study including 76 children with chronic heart disease has demonstrated that galectin-3 is positively associated with the Ross classification score for pediatric heart failure and plays an important role in early diagnosis and prognosis prediction [39]. The studies regarding application of galectin-3 in pediatric patients with heart failure requiring ECLS are quite scarce compared to adult patient populations and all the studies only evaluate VAD patients (Table 2).

## 3. sST2

sST2 is a circulating form of suppression of tumorigenicity-2 (ST2) glycoprotein that is a member of the interleukin 1 receptor family. The ST2 glycoprotein is encoded by the IL1RL1 gene located in the chromosome 2q12. It serves as the receptor for IL-33, an IL-1–like cytokine that can be secreted by living cells in response to cell damage [42]. IL-33 exerts its cardioprotective function by reducing cardiac fibrosis and inflammation [43]. sST2 can eliminate this cardioprotective function by acting as a decoy to IL-33 [43] and thus is considered an indicator of adverse outcome [44] and a prognostic predictor for heart disease without ECLS [45,46,47]. Moreover, a pooled study including 1800 elderly patients who underwent cardiac surgery has demonstrated that a higher sST2 level is a soothsayer for an increased incidence of cardiovascular events or mortality [48]. The prognostic value of sST2 in heart failure may benefit physicians by allowing them a way to identify patients with a high risk of adverse events early in their course of care.

sST2 has been much less studied in ECLS than galactin-3. One study by Tseng et al. [49] showed that the sST2 level was significantly increased in 95% of adult patients, aged 17 to 68 years before VAD implantation (the median sST2 level was 74.2 ng/mL with the normal value defined as <35 ng/mL). sST2 then significantly decreased during VAD support and normalized after 6 months (29.5 ng/mL), with the maximum drop occurring by 3 months (no significant decrease thereafter). They concluded that the high sST2 levels predicted poorer outcomes in patients on conventional treatments and was a consequence of end-stage heart failure. Their data suggest that the sST2 level was a useful parameter to monitor therapy. However, they failed to show whether high sST2 levels at any timepoint can predict outcomes post implantation. Similar to galectin 3, there are limited studies (Table 3) in children and will be discussed in a later section.

## 4. Dynamic Changes of Galectin-3 and sST2 in Adult and Children Undergoing ECLS: Similarities and Differences

**Figure 1:**
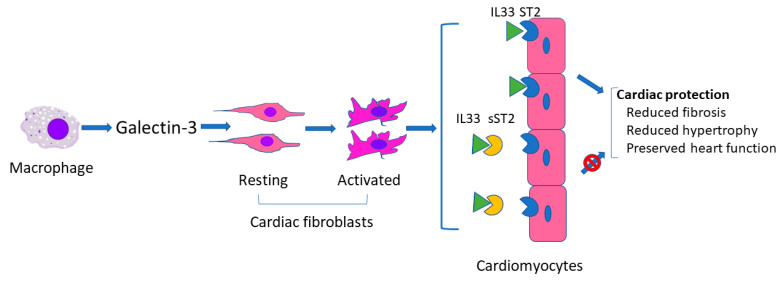
Schematic of possible mechanism of galectin 3 and sST2 in heart failure.

As described above, galectin-3 and sST2 have been used alone or concomitantly as biomarkers in several studies regarding heart failure with or without ECLS [28,31,46]. Galectin-3 and sST2 are similar in that both can reflect severity of myocardial damage (presumably related to fibrosis) to thereby predict prognosis. However, they act differently in the development of heart failure. As shown in Figure 1, in response to cardiac injury, activated macrophages produce galectin-3 which is then thought to regulate phenotypic change of cardiac fibroblasts from the resting to the activated status [50], whereas sST2 binds to IL33 to block the binding of IL33 to ST2 on cardiomyocytes. Binding of IL33 to cardiomyocyte membrane ST2 results in the initiation of IL33/ST2 pathway which then evokes an antihypertrophic and antifibrotic function [51].

**Figure 2:**
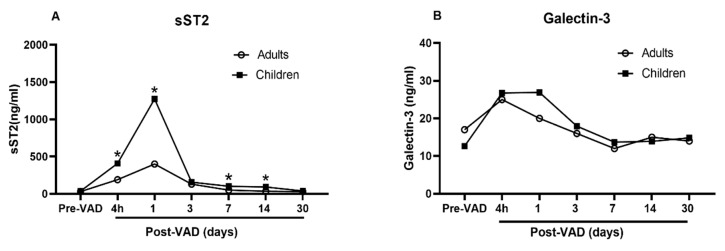
Comparison of sST2 and galectin-3 between adults and children undergoing VAD. Redrawn based upon data reported in [15]. (**A**) Significantly higher circulating level of sST2 in children than in adults at every time points; (**B**) No difference in the circulating level of galectin-3.

Data regarding the similarities and differences of galectin-3 and sST2 between adults and children at baseline and while undergoing ECLS are extremely limited. To date, only one study [16] is available to compare the dynamic changes of galectin-3 and sST2 in adults and children with heart failure requiring VAD. The investigators demonstrated that the galectin-3 and sST2 from adults and children show a similar trend, climbing up one day after VAD implant, and plunging down two days after VAD implant and to baseline levels in 30 days (Figure 2, redrawn based on the data in the study). The circulating level of sST2 is significantly higher in children than in adults at every time points (Figure 2A). In contrast, the circulating level of galectin-3 is not different (Figure 2B). These data indicate differing responses of galectin-3 and sST2 with VAD implant in children compared to adults. The changes of galectin-3 and sST2 in day 1 and 2 may be a result of macrophage activation related to inflammatory processes surrounding surgical implantation of a VAD. Their differences may indicate varying degree of macrophage activation between children and adults.

To the best of our knowledge, no data are available to describe the trend and prognostic value of plasma or serum galectin-3 and sST2 in children or adults undergoing VA-ECMO for cardiac failure. VA-ECMO use is much more common in pediatric patients than VAD implantation compared to adults, and this deficit requires further studies to fill this gap of our knowledge.

## 5. Feasibility of Using Galectin-3 and sST2 as Prognosticators

The general principles to evaluate feasibility of a biomarker include the following: (a) it is easily obtained, (b) highly reproducible, (c) it is biologically plausible and (d) impacts care. Obtaining a blood sample is part of the postoperative routine and does not involve technically complicated procedures. The measurement of plasma or serum galectin-3 and sST2 would not be a significant burden to a current clinical protocol. Second, a biomarker should be scientifically reproducible and financially affordable. The assays for human plasma/serum galectin-3 and sST2 have been commercially available for clinical and research purposes [52]. The stability in vitro, biological variation, and reference values for galectin-3 and sST2 have been previously summarized in a comprehensive review [52] that demonstrates these 2 biomarkers should be clinically reproducible across laboratories. Lastly, studies have highlighted the potential role of galectin-3 and sST2 in the prediction of prognosis in many clinical settings to impact care as discussed above.

Unlike the traditional biomarkers including natriuretic peptides and troponins, sST2 is relatively independent from age, prior diagnosis of HF, body mass index, ischemic type of HF, or atrial fibrillation [53]; galectin-3 is thought to reflect myocardial remodeling and appears to be dynamical biomarker in long-term ECLS. However, galectin-3 is also associated with various fibrotic conditions (liver and lung) [54,55] and this could be a potential confounder in developing treatment strategies.

## 6. Possibility to Use Galectin-3 and sST2 as Indicators to Adjust Medical Regimens or as Therapeutic Targets

Natriuretic peptide-guided therapy in chronic heart failure has been reported in some studies with promising outcomes [56,57,58], whereas other studies have reported uncertain results [59,60]. The controversies suggest inadequate power to draw a conclusion in biomarker-guided treatment for heart failure. Galectin-3 and sST2 have not yet been sufficiently studied in guiding treatment in patients with heart failure who receive pharmacotherapy, not to mention in patients with heart failure who require ECLS.

As described in Figure 1, galectin-3 is an initiator of the inflammation process in heart failure. Targeting galectin-3 may be a potential therapy to improve the outcomes of heart failure. Extracellular and intracellular small-molecule galectin-3 inhibitors (3,3′-Bis-(4-aryltriazol-1-yl) thiodigalactosides [61] and galectin-3 inhibitor compound 2H [62]) have been investigated [63]. The availability of these inhibitors has laid a foundation for further study of a targeted treatment of galectin-3. Interestingly, modified citrus pectin (a dietary supplement) has been used as an inhibiter of galectin-3 to block cardiac injury that is induced by acute kidney injury via the galectin-3 pathway [64] and may provide an easy initial molecule for clinical trials.

sST2 concentrations have been used to identify patients with chronic heart failure who may particularly benefit from β-adrenergic blocker therapy [47]. At cutoff values of sST2 level of 35 ng/mL and with a metoprolol dose of 50 mg daily (defined as a high dose in the study), patients with low sST2/high-dose BB had the lowest cardiovascular event rate (0.53 events); those with low sST2/low-dose β-adrenergic blocker, or high sST2/high-dose β-adrenergic blocker had intermediate outcomes (0.92 and 1.19 events); patients with high sST2 treated with low-dose β-adrenergic blocker had the highest cardiovascular event rate (2.08 events).

In terms of a targeted therapy on sST2 itself, no chemical compound serving as a sST2 antagonist has been reported. Instead, an anti-ST2 mAb has been used to block the interaction between sST2 and IL33 to release free IL33 [65]. The concern is that the anti-ST2 mAb can block the cell membrane ST2 [66] to thereby suppress the IL33/ST2 pathway that is considered cardioprotective.

Neither galectin-3 and/or sST2 has been examined as guides for adjusting medical management for heart failure in pediatric patients, and thus the role of galectin-3 and /or sST2 as a guide to therapeutic decision-making remains to be established. Additionally, within the pediatric patient population, the use of galectin-3 and/or sST2 as a biomarker for risk stratification in children undergoing ECLS with VAD has not been reported, and the impact of VA-ECMO on galectin-3 and sST2 remains unknown.

## 7. Conclusions

Undergoing ECLS creates a complex clinical situation with challenges related to early and accurate prediction of prognosis, particularly in pediatric patients. To distinguish patients who will improve and those who will not early during ECLS is imperative as would not only assist the medical team to formulate an optimal care plan but may also provide a scientific justification to initiate ethical discussions with the patient’s family. Galectin-3 and sST2 have come to prominence as early prognosticators in adult ECLS patients since other biomarkers (BNP [67], NT-proBNP, TnIc, MR-proANP, proADM, and copeptin [14]) have failed to show significance. To discern the complex differences of biomarkers, further studies are needed to investigate the use of a single biomarker (galectin-3 or sST2) versus combined biomarkers (galectin-3, sST2 and/or other markers) which has been done for adult with heart failure but not yet for ECLS patient [40,41,68], and sampling at single time point versus multiple time points in ECLS patients.

Beyond protein biomarkers, circulating microRNAs are emerging as intriguing, predictive biomarkers for heart failure. These microRNAs are attractive candidates due to their known biologic roles in reverse remodeling [69,70] and their ability to discriminate heart failure of different etiologies due to their cell-type specific expression [71]. Akat et al. demonstrated a significant increase in heart-specific circulating microRNAs in patients with advanced heart failure that completely reversed 3 months after initiation of VAD support [72]. This suggests that the decreased levels of circulating microRNAs are associated with favorable outcomes following VAD support. While no data are available to show a link between circulating microRNAs and prognosis of heart failure requiring ECLS, the potential value of circulating microRNAs in predicting ECLS outcomes in the near future should not be overlooked and requires further investigation in pediatric patients as well.

Based on the currently available published data, we expect that the combined galectin-3 and sST2 biomarkers, followed serially, will be beneficial in guiding management of children undergoing ECLS in the future but additional work is needed to identify other novel biomarkers (e.g., microRNAs), and biomarker response to other forms of ECLS (such as VA-ECMO) that may serve to improve the care of the pediatric patient population.

## Figures and Tables

**Table 1:** Identification of heart failure-related biomarkers for patients undergoing ECLS.

Biomarkers	Identification
Brain natriuretic peptide (BNP)	cyclic peptide hormone containing disulfide bridge
NT-proBNP	*N*-terminal fragment of BNP
Troponin	calcium-regulatory protein
Galectin-3	carbohydrate-binding protein with a single carbohydrate recognition domain and a unique N-terminus
MMP-2 and 9	one of member of Matrix metalloproteinases (zinc- and calcium-dependent endopeptidases)
sST2	soluble suppression of tumorigenicity 2
Tissue inhibitors of metalloproteinases-1 (TIMP-1)	protein containing an N- and C-terminal domain of 125 and 65 amino acids, respectively, with each containing three conserved disulfide bonds
Osteopontin	extracellular structural protein
MR-proANP	mid-regional fragment of proANP (ANP precursor)
proADM	long-acting vasodilatory peptide
Copeptin	39-amino-acid glycopeptide and the C-terminal portion of provasopressin

**Table 2:** Application of galectin-3 in adult and pediatric patients with heart failure requiring ECLS.

Reference	Year	Adult/Peds	N =	Population	Major Finding
[17]	2008	Adult	40	VAD	Higher Gal-3 pre implant associated with mortality (n = 15) compared to bridged to transplant (n = 25) (13.4 + 3.6 ng/mL vs. 9.6 + 5.2 ng/mL, *p* < 0.02)
[36]	2013	Adult	175	VAD	Higher Galectin-3 levels (>17 ng/mL) increased mortality for low/medium risk VAD patients
[40]	2015	Adult	25	VAD	Gal-3 remains elevated after continuous flow VAD placed
[41]	2015	Adult	37	VAD	Gal-3 decreases during LVAD support
[35]	2016	Adult	57	VAD	Galectin-3 levels >30 ng/mL are associated with lower survival post-LVAD placement (76.5% versus 95.0% at 2 years, *p* = 0.009)
[15]	2018	Both	7 adult 12 pediatric	VAD	Children similar Galectin-3 levels as adults post VAD

**Table 3:** Application of sST2 in adult and pediatric patients with heart failure requiring ECLS.

Reference	Year	Adult/Peds	N =	Population	Major Finding
[41]	2015	Adult	37	VAD	sST2 decreases during LVAD support
[48]	2018	Adult	38	VAD	sST2 elevated in VAD patients and normalized after 6 months; not predictive of outcomes
[15]	2018	Both	7 adult 12 pediatric	VAD	sST2 level in children is different than adults following VAD implant

## Data Availability

Not applicable.
